# Breast cancer cell-derived fibroblast growth factors enhance osteoclast activity and contribute to the formation of metastatic lesions

**DOI:** 10.1371/journal.pone.0185736

**Published:** 2017-10-02

**Authors:** Kelly Aukes, Cynthia Forsman, Nicholas J. Brady, Kristina Astleford, Nicholas Blixt, Deepali Sachdev, Eric D. Jensen, Kim C. Mansky, Kathryn L. Schwertfeger

**Affiliations:** 1 Department of Lab Medicine and Pathology, University of Minnesota, Minneapolis, Minnesota, United States of America; 2 Microbiology, Cancer Biology and Immunology Graduate Program, University of Minnesota, Minneapolis, Minnesota, United States of America; 3 Developmental and Surgical Science, School of Dentistry, University of Minnesota, Minneapolis, Minnesota, United States of America; 4 Department of Genetics, Cell Biology, and Development, University of Minnesota, Minneapolis, Minnesota, United States of America; 5 Masonic Cancer Center, University of Minnesota, Minneapolis, Minnesota, United States of America; 6 Department of Medicine, Division of Hematology, Oncology and Transplantation, University of Minnesota, Minneapolis, Minnesota, United States of America; 7 Department of Diagnostic and Biological Science, School of Dentistry, University of Minnesota, Minneapolis, Minnesota, United States of America; 8 Center for Immunology, University of Minnesota, Minneapolis, Minnesota, United States of America; Charles P. Darby Children's Research Institute, 173 Ashley Avenue, Charleston, SC 29425, USA, UNITED STATES

## Abstract

Fibroblast growth factors (FGFs) and their receptors (FGFRs) have been implicated in promoting breast cancer growth and progression. While the autocrine effects of FGFR activation in tumor cells have been extensively studied, little is known about the effects of tumor cell-derived FGFs on cells in the microenvironment. Because FGF signaling has been implicated in the regulation of bone formation and osteoclast differentiation, we hypothesized that tumor cell-derived FGFs are capable of modulating osteoclast function and contributing to growth of metastatic lesions in the bone. Initial studies examining FGFR expression during osteoclast differentiation revealed increased expression of FGFR1 in osteoclasts during differentiation. Therefore, studies were performed to determine whether tumor cell-derived FGFs are capable of promoting osteoclast differentiation and activity. Using both non-transformed and transformed cell lines, we demonstrate that breast cancer cells express a number of FGF ligands that are known to activate FGFR1. Furthermore our results demonstrate that inhibition of FGFR activity using the clinically relevant inhibitor BGJ398 leads to reduced osteoclast differentiation and activity *in vitro*. Treatment of mice injected with tumor cells into the femurs with BGJ398 leads to reduced osteoclast activity and bone destruction. Together, these studies demonstrate that tumor cell-derived FGFs enhance osteoclast function and contribute to the formation of metastatic lesions in breast cancer.

## Introduction

While the 5-year survival rate for breast cancer patients has increased to 89% overall, the 5-year survival rate for patients diagnosed with metastatic breast cancer remains low at 24% [1]. Breast cancer metastasizes to several sites including lung, liver, brain and bone, with bone being the preferred site [2]. Bone micrometastases are present in 30% of patients diagnosed with stage I, II and III (non-metastatic) breast cancer and the presence of bone micrometastasis is an independent predictor of poor outcome [3]. Over 70% of advanced breast cancer patients develop overt bone metastasis emphasizing the clinical importance of this phenomenon. Bone metastases are not only severely painful, but they are associated with bone fracture, both of which lead to significant decrease in quality of life. There are limited therapeutic options for managing the clinical impact of bone metastases, other than palliative therapies. Thus, there is an urgent need for developing novel therapies that can be used to help limit the growth and deleterious sequelae of breast tumor lesions localized to bone.

Breast cancers can be subdivided into three clinically distinct subtypes based on the expression of specific receptors (estrogen receptor (ER)/progesterone receptor (PR) positive, Her2/Neu or triple negative), all of which have the capacity to metastasize to the bone [4]. Current therapies for overt metastasis include chemotherapy to reduce tumor growth and bisphosphonates or denosumab to limit bone resorption [5]. Bone pain is currently managed by analgesics or radiation, however these treatments have no beneficial effect on bone fracture. As with any metastasis, breast cancer cells entering the bone must not only adapt to this new tissue, but they also actively condition the bone microenvironment to assist in the expansion and further progression of the metastatic lesion.

The bone is comprised of multiple cell types, including osteoblasts (mesenchymal-derived cells responsible for synthesizing bone) and bone degrading osteoclasts, which arise from hematopoietic derived stem cells [5]. Bone homeostasis and normal function result from the proper balance of osteoblasts and osteoclasts. To maintain this balance in the adult skeleton, osteoblasts produce the cytokine receptor activator of NF-κB ligand (RANKL), a factor required to promote osteoclast differentiation [5]. During bone resorption by the osteoclast, growth factors embedded in the bone matrix are released and help stimulate the differentiation of osteoblasts and a corresponding increase in RANKL production [5]. Breast cancer bone metastases are primarily associated with excess bone degradation (osteolysis) suggesting that tumor cells are capable of enhancing osteoclast differentiation and/or activity [2]. Therefore, is it critical to identify key components of this complex regulatory network that can be exploited for successful therapeutic management of breast cancer patients.

The fibroblast growth factor (FGF) family consists of 22 FGF ligands that bind and activate FGF receptors (FGFRs), which are transmembrane receptor tyrosine kinases [6]. The ligands typically act in a paracrine manner, in which they are secreted from one cell type and activate their target FGFRs on a different cell type. There are four FGFR genes (FGFR1-4), and FGFR1-3 are alternatively spliced in the extracellular domain to generate the iiib and iiic receptor isoforms. These alternative splicing events result in altered specificity for the FGF ligands. In addition, these isoforms exhibit cell-type specific expression; the iiic isoforms are typically expressed in mesenchymal-derived cells and the iiib isoforms are typically expressed in non-mesenchymal cells [6]. FGFs and their receptors have been implicated in skeletal development. Specifically, *Fgfr2iiib-/-* mice exhibit pronounced skeletal defects, although the mechanisms have not been defined [7]. In addition, *Fgfr2iiic* -/- mice have defects in bone formation partially due to misregulation of osteoblasts [8]. Furthermore, exogenous FGFs have been shown to promote osteoclast [9] and osteoblast [10] differentiation and function. However, the effects of FGFR activation in the tumor microenvironment of bone metastatic lesions have not been examined.

In addition to regulating normal developmental processes, alterations in the FGF/FGFR axis contribute to growth and progression of a number of cancers, including breast cancer [11]. Specifically, the growth and malignant progression of triple negative tumors are linked to increased production of FGF ligands and subsequent aberrant activation of FGFR [12]. Not surprisingly, FGFR inhibitors are currently being evaluated in clinical trials for patients with primary and metastatic breast cancer [11, 13, 14]. Because FGFR inhibitors are already in the clinical setting, experimental support for the importance of this pathway in the growth and/or maintenance of metastatic bone lesions in breast cancer could lead to rapid translation of these findings to clinical applications. In this study, we demonstrate that tumor cell derived factors are able to enhance osteoclast differentiation and activity in part through activation of FGFR in osteoclasts. Furthermore, we demonstrate that FGFR inhibition leads to reduced osteoclast activity and bone degradation *in vivo*. These findings support further analysis of FGFR inhibitors in the context of breast cancer bone metastasis.

## Material and methods

### Ethics

The use and care of the mice was reviewed and approved by the University of Minnesota Institutional Animal Care and Use Committee, IACUC protocol numbers 150732820A and 160133265A. Euthanasia was performed by CO_2_ inhalation.

### Cell lines and cell culture

MCF10A and MDA-MB-231 cells were obtained from ATCC and maintained under recommended conditions. MDA-MB-231-BoM-1833 (BoM-1833) cells were obtained from Dr. Joan Massague (Howard Hughes Medical Institute, Memorial Sloan Kettering, New York, NY) and maintained as described [15]. To harvest conditioned media, cells were grown to confluency and incubated in serum free media for 24 hours prior to collection. HC-11/R1 cells were obtained from Dr. Jeffrey Rosen (Baylor College of Medicine, Houston, TX) and maintained as described [16]. For *in vitro* analysis of cell survival, BoM-1833 cells and HC-11/R1 cells were treated with the indicated amounts of BGJ398 and 30 nM B/B (to activate inducible FGFR1 in HC-11/R1 cells only) prior to assessment of survival by MTS assay. CMG14-12 cells were obtained from Dr. Sunao Takeshita (Nagoya City University, Nagoya, Japan).

### Antibodies and chemicals

Total and phosphorylated p38 (9212, Antibody ID# AB330713 and 9211, Antibody ID# 331641) are polyclonal antibodies produced against sequence of human p38 MAPK or synthetic peptide corresponding to Thr180 and Tyr182 of human p38 MAPK, ERK and pERK (9102, Antibody ID# AB330744 and 9101, Antibody ID# AB331646) are polyclonal antibodies produced against carboxy terminus of p42/44 MAPK or synthetic peptide corresponding to Thr202 and Tyr204 of human p42/44 MAPK, AKT (4691, Antibody ID#AB915783) is a monoclonal antibody raised against carboxy terminus sequence of mouse AKT, pAKT (4058, Antibody ID#331168) is a monoclonal antibody produced against a synthetic peptide around residues of Ser473 of the mouse sequence, alpha-tubulin (2144, Antibody ID#2210548), a polyclonal antibody raised against the sequence of human alpha-tubulin, beta-tubulin (2146, Antibody ID#2210545) is a polyclonal antibody produced using a synthetic peptide against human β-tubulin and FGFR1 (3472, Antibody ID#10691847) is a polyclonal antibody raised against amino terminal peptide of human FGFR1 antibodies. All antibodies used in this study were obtained from Cell Signaling Technologies. All antibodies were used at a 1:1,000 dilution in Western blots. BGJ398 was obtained from Selleckchem.

### Harvesting of bone marrow for osteoclast cultures

Primary bone marrow macrophages were harvested from the femurs and tibiae of 4-week-old C57Bl/6 mice as previously described [17]. Briefly, the femurs and tibiae were dissected and adherent tissue was removed. The ends of the bones were cut and the marrow was flushed from the inner compartments. Red blood cells were lysed from the flushed bone marrow tissue with RBC lysis buffer (150 mM NH_4_Cl, 10 mM KHCO_3_, 0.1 mM EDTA, pH7.4) and the remaining cells were plated on 100 mm plates and cultured overnight in osteoclast medium (phenol red-free Alpha-MEM (Gibco) with 5% fetal bovine serum (Hyclone), 25 units/mL penicillin/streptomycin (Invitrogen), 400 mM L-Glutamine (Invitrogen), and supplemented with 1% CMG 14–12 culture supernatant containing M-CSF. The non-adherent cell population, including osteoclast precursor cells, was then carefully separated and re-plated at approximately 2x10^5^ cells/cm^2^ in a 12 well plate with osteoclast medium supplemented with 1% CMG 14–12 culture supernatant containing M-CSF. Two days later, this medium was replaced with medium containing 1% CMG 14–12 culture supernatant, 10ng/mL RANKL (R&D Systems) and 50% serum free medium (growth media), or serum free medium collected from 10A, MDA-MB 231 or BoM-1833 cells to stimulate osteoclast differentiation. For inhibition of FGF signaling osteoclasts were treated with conditioned medium as described above with either DMSO (vehicle) or 40 nM BGJ398.

### Harvesting RNA

Quantitative real-time PCR was performed using the MyiQ Single Color Real-Time PCR Detection System (Biorad). RNA was harvested from cells using Trizol Reagent (Ambion, Life Technologies) and quantified using UV spectroscopy. cDNA was prepared from 1 μg RNA using the iScript cDNA Synthesis Kit (Biorad) as per the manufacturer’s protocol. Experimental genes were normalized to *Hprt*. *Hprt* (Forward) GAGGAGTCCTGTTGATGTTGCCAG;(Reverse) GGC TGGCCTATAGGCTCATAGTGC; *c-Fos* (Forward) CCAAGCGGAGACAGATCA ACT;(Reverse) TCCAGTTTTTCCTTCTCTTTCAGCAGA; *Nfatc1* (Forward)TCATCCTGTCCAACACCAAA;(Reverse) TCACCCTGGTGTTCTTCCTC; *Dc-stamp* (Forward) CAGACTCCCAAATGCTGGAT;(Reverse) CTTGTGGAGGAACCTAAGCG; *Oscar* (Forward) TCATCTGCTTGGGCATCATA; (Reverse) ACAAGCCTGACAGTGTGGTG; *Itgb3* (forward) CTGGTAAAACGCGTGAAT;(Reverse) CGGTCATGAATGGTGATGAG and *cathepsin K* (Forward) AGGGAAGCAAGCACTGGATA; (Reverse) GCTGGCTGGAAT CACATCTT; *Fgf1* (Forward) ACACCGACGGGCTTTTATACG; (Reverse) CCCATTCTTCTTGAGGCCAAC; *Fgf19* (Forward) CTACAATGTGTACCGATCCGAG; (Reverse) TCCGGTGACAAGCCCAAATG; *Fgf21*(Forward) ATGGATCGCTCCACTTTGACC; (Reverse) GTGTGGGGACTTGTTCCCT; *Fgf23* (Forward) CAGAGCCTATCCCAATGCCTC; (Reverse) GGCACTGTAGATGGTCTGATGG; *Fgfr1iiib* (Forward) CCTTACACACATACTCCCCGC; (Reverse) CTTGCCGTATGTCCAGATCC; *Fgfr1iiic* (Forward) CTTGCCGTATGTCCAGATCC; (Reverse) TCCGTAGATGAAGCACCTCC; *Fgfr2iiib* (Forward) ACTGTCCTGCCCAAACAGC; (Reverse) GCAGGCGATTAAGAAGACCC; *Fgfr2iiic* (Forward) TGCATGGTTGACAGTTCTGC; (Reverse) GCAGGCGATTAAGAAGACCC; *Fgfr3iiib* (Forward) GAAGCACGTGGAAGTTGAACG; (Reverse) CCACATTCTCACTGATCCAGG; *Fgfr3iiic* (Forward) GAAGCACGTGGAAGTGAACG (Reverse) TCCTTGTCGGTGGTGTTAGC; *Fgfr4* (Forward) CCACACTTTCCATCACCAGG; (Reverse) TACCATCCACTGGCTCAAGG.

### Resorption assay

Primary bone marrow macrophages were plated on Osteo Assay Surface plates (Corning) at a density of 1x10^5^ cells per well. Cells were allowed to fully differentiate in the presence of 1% CMG 14–12 conditioned media, RANKL and conditioned medium as described for osteoclast cultures. The medium was completely removed on day 5 and 100μL/ well of 10% bleach or TRAP stain was added and allowed to incubate at room temperature for 5 minutes. The bleach solution or TRAP solution was then aspirated and the wells were washed twice with 150μL of dH_2_O. The plate was then allowed to air dry completely at room temperature for 3–5 hours. The wells were observed under 4x objective for the formation of demineralization and images were captured with light microscopy. Images were measured and analyzed using NIH ImageJ.

### TRAP stain

Primary osteoclasts were fixed with 4% paraformaldehyde (PFA) and washed with PBS. The cells were then stained for tartrate resistant acid phosphatase (TRAP) expression (0.05M acetate buffer; 0.03M sodium tartrate; 100 μg/mL Napthol AS-MX phosphate; 0.01% Triton X-100; 0.3 mg/mL Fast Red Violet LB stain) for approximately 10 minutes at 37°C [17]. Cells were then observed and captured with light microscopy and the measurements were analyzed using NIH ImageJ.

### Quantitating nuclei

TRAP-stained multinuclear osteoclasts were labeled with DAPI to visualize nuclei. Images of cells were captured with light and fluorescence microscopy and total nuclei were quantitated with NIH ImageJ. To calculate number of nuclei per cell, DAPI images were overlaid with TRAP stained images to calculate number of nuclei per cell in TRAP positive cells containing 3 or more nuclei.

### Immunoblotting

Cell protein lysates were harvested from osteoclasts in modified RIPA buffer (50mM Tris pH 7.4, 150mM NaCl, 1% IGEPAL, 0.25% sodium deoxycholate, 1mM EDTA) supplemented with Halt Protease & Phosphatase Inhibitor Cocktail (Thermo Scientific). Lysates were cleared by centrifugation at 12,000X*g* at 4°C. Proteins were resolved by SDS-PAGE and transferred to PVDF membrane (Millipore). HRP-conjugated anti-rabbit (NA-934, Antibody ID# AB772206) was incubated at 1:10,000 with membranes, washed, and incubated with Advansta-Western Bright Sirius detection agent.

### MTS assay

BoM-1833 cells and HC-11/R1 cells were plated at 10,000 cells/well in a 96-well plate. After 24 hours, BGJ398 dissolved in DMSO was added at the indicated concentrations. 30 nM B/B homodimerizer (Clontech) was added to the HC-11/R1 cells to activate FGFR1. Plates were incubated for 72 hours prior to addition of MTS reagent per manufacturer’s instructions (Promega).

### Tumor induction, micro computed tomography and bone morphometric analysis

1x10^5^ BoM-1833 cells were injected directly into the femurs of athymic mice and maintained for 21 days. Mice were treated with 30 mg/kg once daily by oral gavage in DMSO/polyethylene glycol 300 or solvent alone. Samples were scanned post-mortem in air using the XT H 225 micro-CT machine (Nikon Metrology Inc., Brighton, MI, USA) with an isotropic voxel size of 6.7 μm. The scan settings were 90 kV, 90 μA, 720 projections, 2 frames per projection, and an integration time of 708 ms. CT Pro 3D (Nikon metrology, Inc., Brighton, MI, USA) was used to make 3D reconstructions, and VGStudio MAX 2.1 (Volume Graphics GmbH, Heidelberg, Germany) was used to convert reconstructions into BMP datasets. Morphometric analysis was completed with the SkyScan CT-Analyser (CTAn) software (Bruker micro-CT, Belgium) according to Bruker micro-CT’s Method Note 2. Method Note 8 was used to select cortical and trabecular regions of interest in an automated manner. All 3D models were created with the CT-Volume (CTVol) software (Bruker micro-CT, Belgium).

### ELISAs

Serum was harvested at time of euthanasia from animals at 3 months of age and subjected to ELISA as per manufacturer’s protocol. Bone resorption was quantitated with CTX (RatLaps EIA IDS), bone formation was quantitated with P1NP (Rat/Mouse P1NP EIA kit, IDS), and osteoclast number was quantitated with TRAP (Mouse TRAP ELISA, IDS) ELISAs.

## Results

### Expression levels of FGFR1 increase during osteoclastogenesis

**Fig 1:**
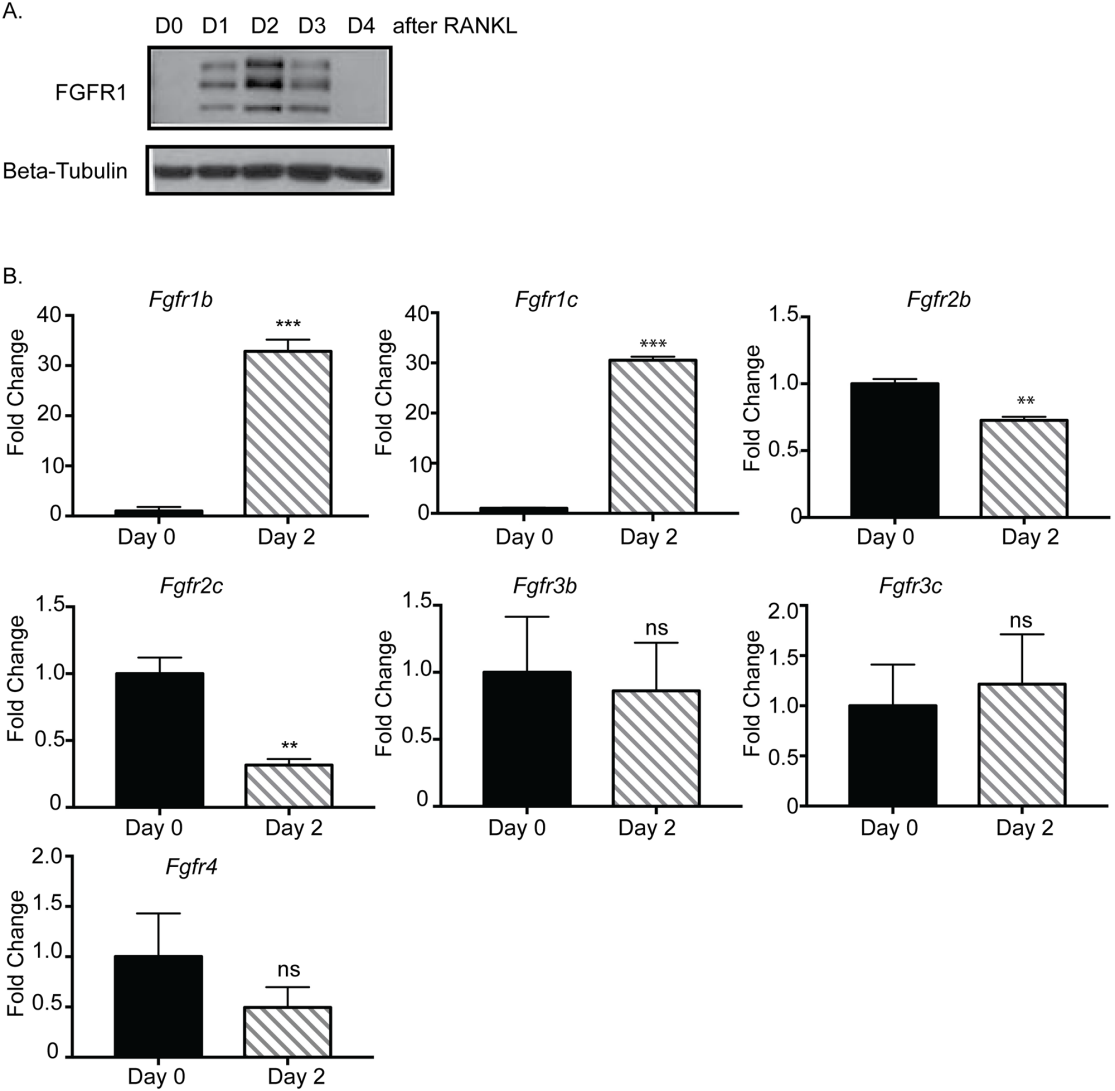
FGF receptor expression in osteoclasts. Bone marrow macrophages were cultured from C57Bl/6 mice in M-CSF and/or RANKL (10 ng/mL) for indicated times. (A) Western blot of osteoclast lysates cultured in M-CSF (day 0) or M-CSF and RANKL (day 1–4). Western blot was probed with an antibody recognizing FGFR1 (Cell Signaling) or β-tubulin (Cell Signaling). (B) Measurement of *Fgf* receptor expression in osteoclasts at day 0 (M-CSF only) or day 2 (M-CSF and RANKL for 48 hours) by qPCR. Experiments were done at least three times and values represent the mean ± SD. ns = not significant **p<0.01, ***p<0.0001 comparing day 0 vs. day 2.

Published studies have demonstrated that FGFRs are critical for bone development [18–20]. FGFR expression levels during osteoclast differentiation have not been previously examined, although Chikazu et al. demonstrated that mature osteoclasts express only FGFR1 [21]. Therefore, initial studies were performed to examine FGFR expression in primary osteoclasts during differentiation. Bone marrow-derived cells were treated with RANKL to induce differentiation and were harvested at days 0–4 of differentiation. FGFR expression was examined by immunoblot analysis using an anti-FGFR1 antibody. FGFR1 expression was not detectable at day 0, but increased by day 1 of differentiation, which then peaked at day 2 and returned to undetectable levels by day 4 of differentiation (Fig 1A, S1 Fig). Multiple reactive bands were observed, likely due to glycosylation of FGFR, which often leads to the presence of more than one band by immunoblot analysis [22]. To determine whether this increase was due to transcriptional regulation of *Fgfr1*, further studies were performed using qRT-PCR analysis of gene expression of the different FGFR isoforms. Expression levels of both *Fgfr1iiib* and *Fgfr1iiic* were significantly enhanced at day 2 of osteoclastogenesis. In contrast, *Fgfr2iiib* and *Fgfr2iiic* expression levels were reduced and *Fgfr3iiib*, *Fgfr3iiic* and *Fgfr4* were detectable but not significantly altered (Fig 1B).

### Treatment of osteoclasts with conditioned medium from breast cancer cells induces activation of FGFR-regulated signaling pathways

**Fig 2:**
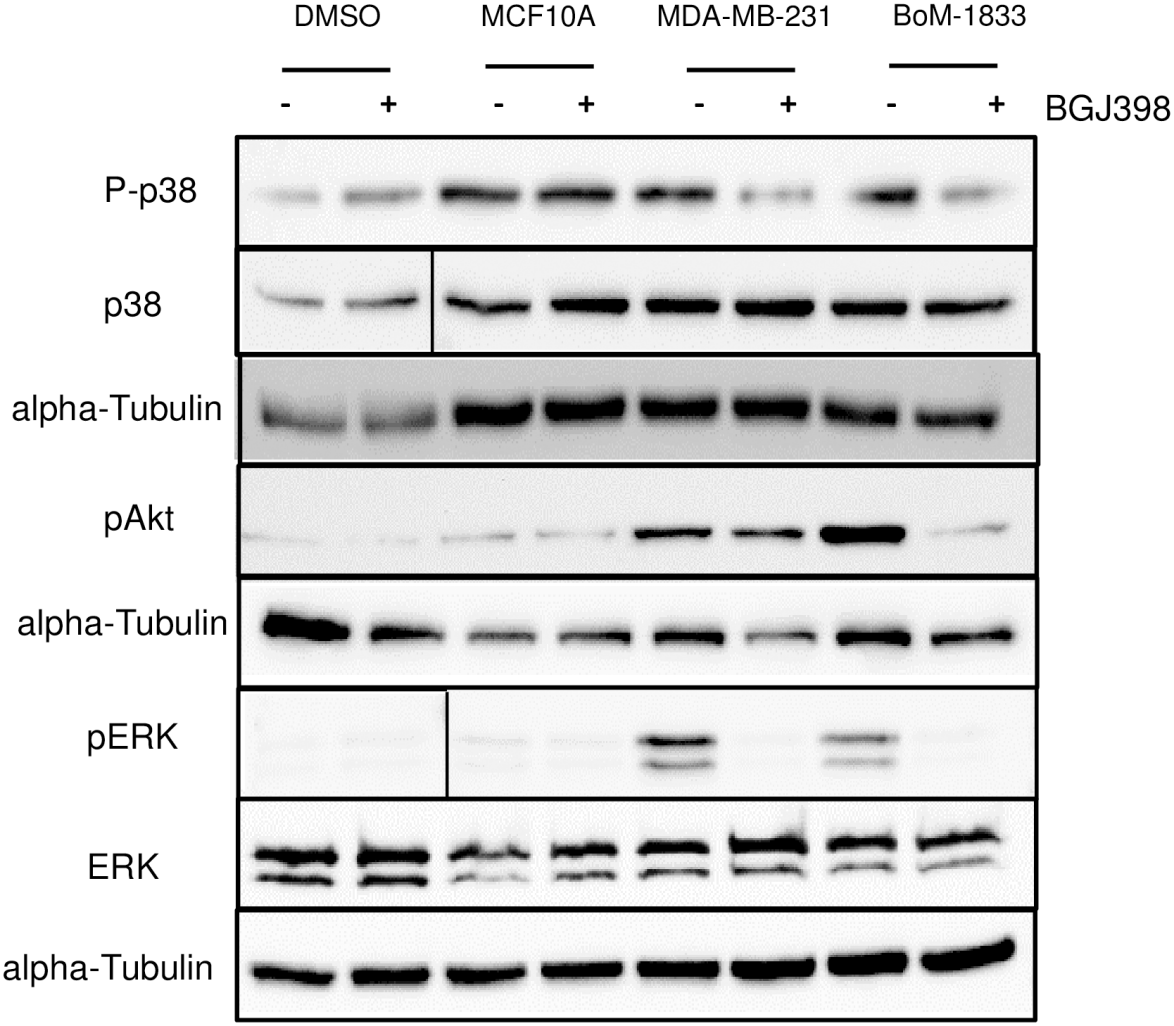
Activation of FGF signaling pathways in osteoclasts. Representative western blot of day 2 osteoclasts were treated with conditioned media from MCF10A, MDA-MB-231 or BoM-1833 cells. Cell lysates were harvested and analyzed for phosphorylated and total p38, ERK, Akt. alpha-tubulin was used as a loading control for protein lysates.

Based on the results that osteoclasts express FGFR1 during osteoclastogenesis, further studies were performed to determine whether conditioned medium collected from breast cancer cells was capable of activating FGFR-induced signaling pathways in osteoclasts. For these studies, serum-free conditioned medium samples were obtained from MCF10A, MDA-MB-231 and MBA-MD-231-BoM-1833 (referred to as BoM-1833) cells. MCF10A cells represent immortalized but non-transformed breast epithelial cells and MDA-MB-231 cells are a triple negative human breast cancer cell line, from which the BoM-1833 cells were derived based on their propensity to seed in the bone [15]. Primary bone marrow derived osteoclasts were treated with conditioned medium for 10 minutes at day 2 of differentiation, which is the peak of FGFR1 expression (Fig 1). Activation of canonical FGFR signaling pathway factors were examined including p38, Akt and ERK by immunoblot analysis. All three cell lines were found to activate p38 whereas high levels of pAkt and pERK were observed only in the osteoclasts treated with medium collected from MDA-MB-231 and BoM-1833 cells (Fig 2, S2 Fig). In contrast, conditioned medium from MCF10A cells did not activate pAkt or ERK, suggesting that non-transformed cells do not produce sufficient levels of soluble factors that activate these signaling pathways in osteoclasts. To determine whether activation of these signaling molecules required FGFR activity, osteoclasts were treated with the FGFR inhibitor BGJ398 at a concentration of 40 nM, which is within the range of FGFR-specificity [23], in conjunction with conditioned media samples. The activation of p38 by MCF10A conditioned medium was not affected by the presence of BGJ398, suggesting that other factors in MCF10A medium activate this pathway (Fig 2). However, treatment of osteoclasts with BGJ398 reduced activation of p38 and pERK by both MDA-MB-231 and BoM-1833 cells and pAkt by the BoM-1833 cells (Fig 2), suggesting that FGFR activity contributes to activation of these pathways in osteoclasts following exposure to tumor cell conditioned media.

### FGF ligand expression in BoM-1833 cells

**Fig 3:**
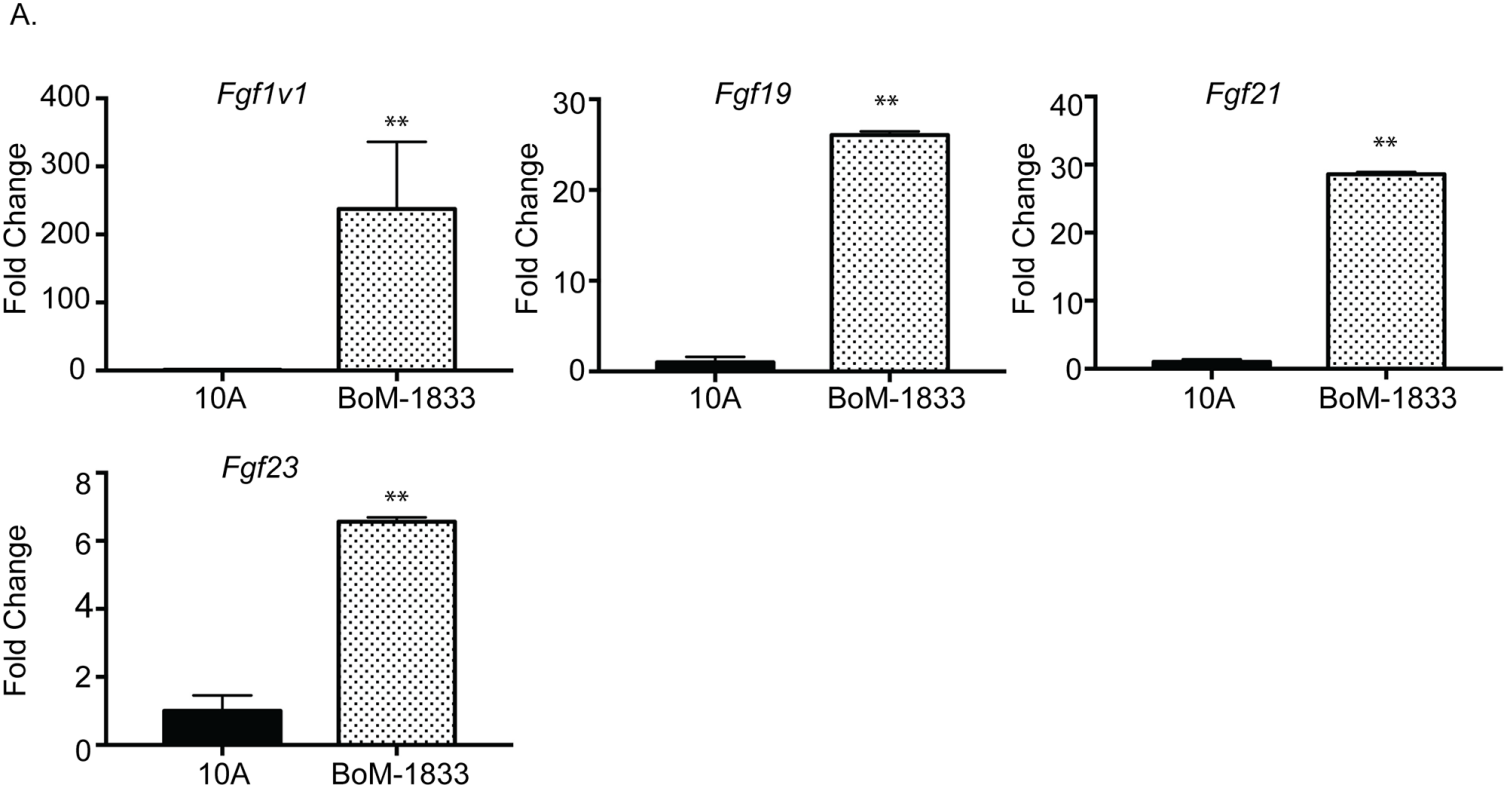
FGF ligand expression in MCF10A and BoM-1833 cells. Real time qPCR of FGF ligands. Experiments were done at least three times and values represent the mean ± SD **p<0.01, comparing MCF10A vs. BoM-1833 conditioned medium.

Based on these results, we further assessed FGF ligand expression in MCF10A and BoM-1833 cells to determine whether BoM-1833 cells secrete higher levels of FGF ligands compared with non-transformed epithelial cells. qRT-PCR analysis was performed to assess gene expression of soluble FGFs that are known to preferentially activate FGFR1, including *Fgf1*, *Fgf2*, *Fgf4*, *Fgf5*, *Fgf6*, *Fgf19*, *Fgf21 and Fgf23*. *Fgf4*, *Fgf5* and *Fgf6* were not detectable by qRT-PCR (data not shown). Analysis of expression levels of the other FGF ligands revealed increased expression of *Fgf1*, *Fgf19*, *Fgf21* and *Fgf23*, and reduced expression of *Fgf2* in BoM-1833 compared with MCF10A cells (Fig 3A and data not shown). These findings demonstrate an overall increase in expression of FGF ligands by BoM-1833 cells compared with non-transformed MCF10A cells. Because these ligands are all capable of activating FGFR1 [24], it is feasible that they may all contribute to FGFR1 activation in osteoclasts.

### Tumor cells enhance osteoclast function in an FGFR-dependent manner

**Fig 4:**
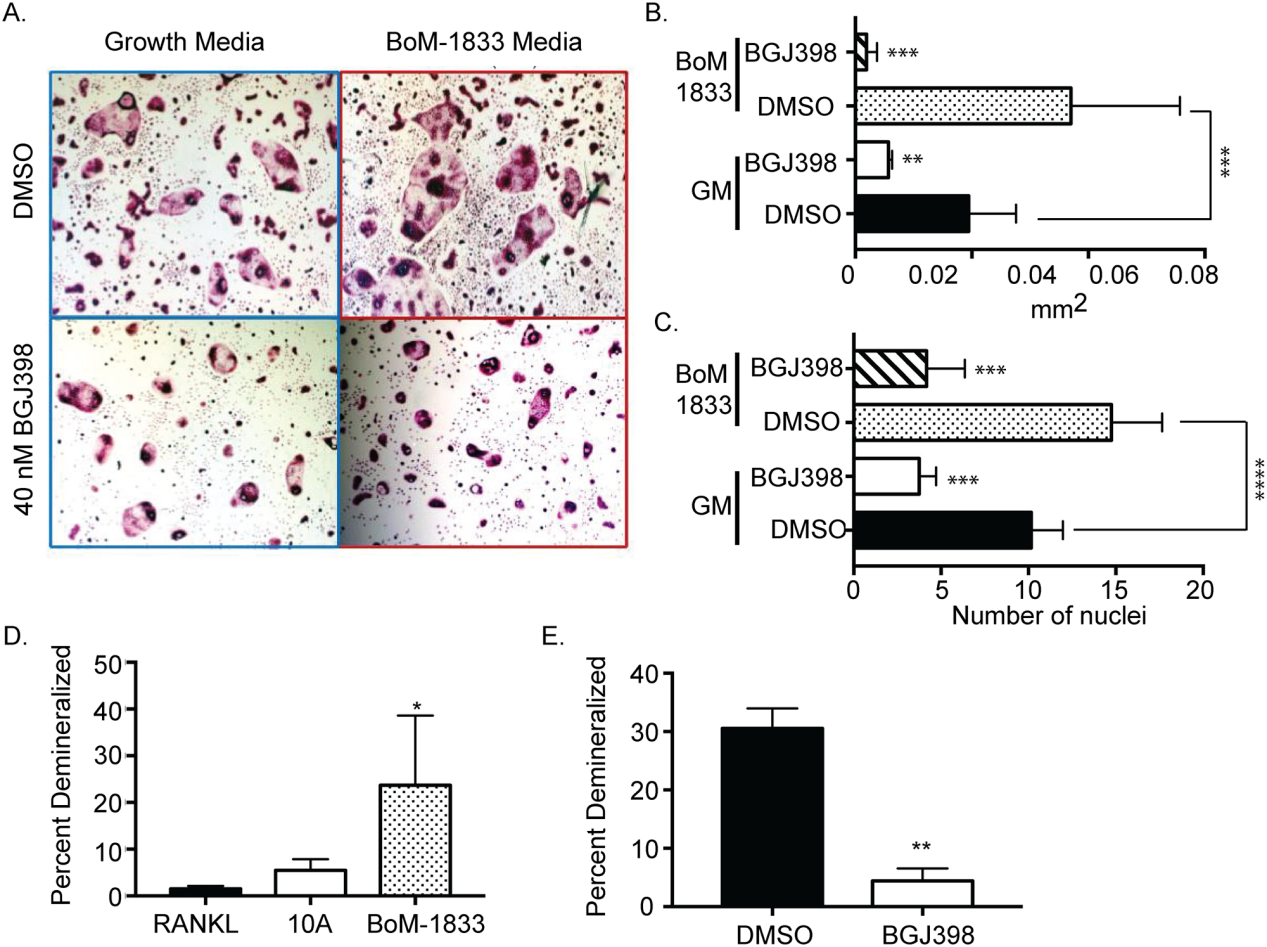
Osteoclast differentiation is enhanced by FGF from BoM-1833 medium. BMMs were harvested from C57Bl/6 mice and differentiated in the presence of M-CSF and RANKL ± BoM-1833 conditioned medium ± BGJ398. (A) Representative TRAP images of different treatments (B) Quantification of osteoclast size (C) Quantification of nuclei per cell (D) Quantification of percent area resorbed by ostoeclasts in growth, MCF10A or BoM-1833 conditioned medium. (E) Quantification of percent area resorbed by osteoclasts in BoM-1833 conditioned medium ± BGJ398. Experiments were done at least three times and values represent the mean ± SD. ns = not significant * p<0.05, **p<0.01, ***p<0.0001.

To determine whether enhanced FGFR activity affects osteoclast differentiation in response to tumor cell conditioned media, bone marrow cells were cultured with M-CSF and RANKL and once multinuclear cells appeared, the cells were stained with TRAP for visualization and assessed by light microscopy. Treatment of osteoclasts with conditioned medium from BoM-1833 cells led to the larger osteoclasts as measured by area and number of nuclei per osteoclast, and this was significantly altered compared with osteoclasts differentiated with standard growth medium (Fig 4A–4C). To determine whether FGFR activity contributes to osteoclastogenesis, osteoclasts were treated with either standard growth medium or conditioned medium obtained from BoM-1833 cells in the presence of BGJ398. As shown in Fig 4B and 4C, the addition of BGJ398 reduced both osteoclast area and median nuclei numbers in both conditions. Similar to the effects we measured with osteoclast differentiation, osteoclasts treated with conditioned medium from BoM-1833 cells demineralized more of a calcium phosphate coated well and the presence of BGJ398 significantly reduced the increased demineralization (Fig 4D and 4E). Taken together, these studies suggest that FGFR inhibition reduces osteoclastogenesis, although this is not restricted to conditions under which tumor cell-derived factors are present.

### Alterations in osteoclast gene expression by tumor conditioned media

**Fig 5:**
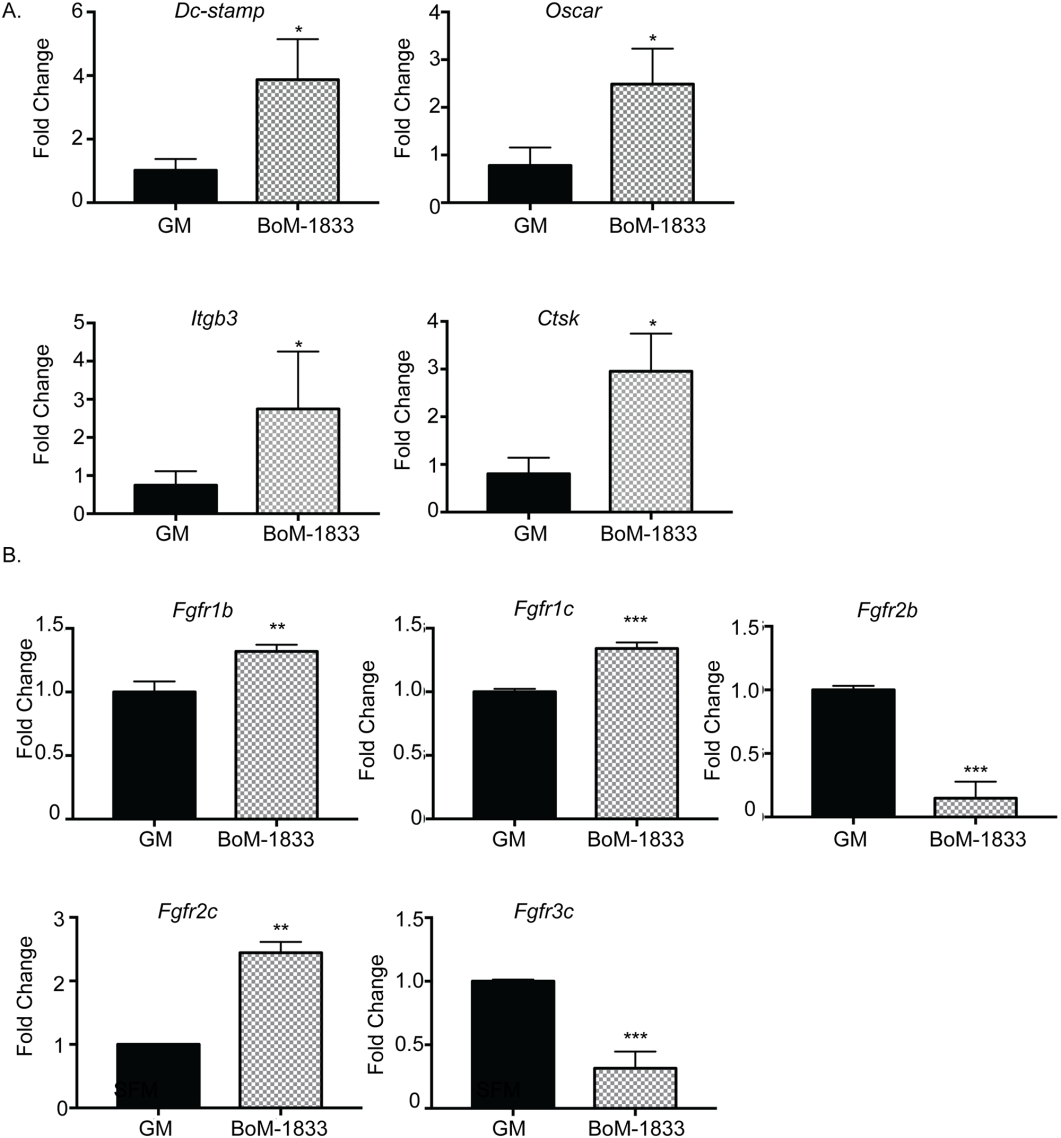
FGF receptor expression stimulated by BoM-1833 media in osteoclasts. BMMs were harvested from C57Bl/6 mice and differentiated in the presence of M-CSF and RANKL for 5 days in the presence of serum free media (SFM) or BoM-1833 media. (A) Real time qPCR of *Dc-stamp*, *Oscar*, *Itgb3*, and *Ctsk* as markers of osteoclast differentiation. (B) Expression of *Fgf* receptors was measured by qPCR. Experiments were done at least three times and values represent the mean ± SD. * p<0.05, **p<0.01, ***p<0.0001 comparing day SFM vs. BoM-1833 medium treated osteoclasts.

Further studies were performed to determine whether conditioned medium from BoM-1833 cells altered genes necessary that could potentially mediate the responses observed, including genes associated with osteoclast differentiation and FGFRs, which could modulate sensitivity to FGF treatment. Osteoclasts were treated with standard growth medium or conditioned medium from BoM-1833 for 5 days. We detected no change in expression of the transcription factors *c-Fos* or *Nfatc1* (data not shown); however, we did detect significant increases in expression of *Dc-stamp*, *Oscar*, *Itgb3* and *Cathepsin K* genes necessary for osteoclast fusion and activity [25] (Fig 5A).

In addition to increased expression of FGF ligands by breast cancer cells, it is also possible that exposure of osteoclasts to tumor cell conditioned medium increases expression of FGFRs in osteoclasts, which may enhance the sensitivity of these cells to FGF ligands. To assess this possibility, FGFR expression was examined in osteoclasts following exposure to conditioned media from BoM-1833 cells. Interestingly, increased expression levels of *Fgfr1iiib*, *Fgfr1iiic* and *Fgfr2iiic* whereas decreased expression of *Fgfr2iiib* and *Fgfr3iiic* were observed (Fig 5B). These results demonstrate that tumor cell media may modulate expression levels of FGFRs in an isoform dependent manner and that although the magnitude of change in *Fgfr1* isoforms is minimal, *Fgfr2* and *Fgfr3* isoforms are altered, possibility leading to differential sensitivity of osteoclasts to different ligands.

#### Treatment of tumor bearing mice with BGJ398 leads to reduced osteoclast activity

**Fig 6:**
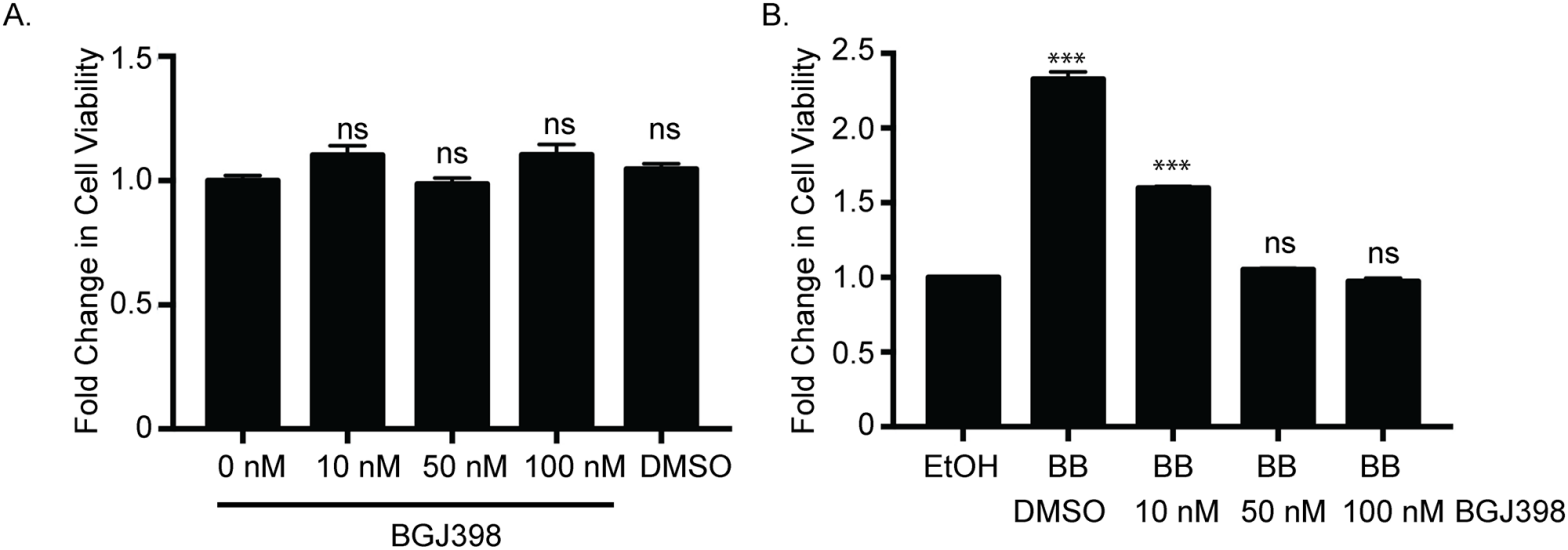
BGJ398 does not affect proliferation of BoM-1833 cells. (A) MTS assay of BoM-1833 cells treated with increasing concentrations of BJG398. (B) MTS assay of HC-11/R1 cells treated with increasing concentrations of BJG398. ns = not significant, ***p<0.0001 comparing DMSO vs. BGJ398 treated (A) or EtOH vs. BB treated (B).

Previously published studies have suggested that parental MDA-MB-231 cells are not responsive to FGFR inhibition [12]. Because the effects of FGFR inhibition on BoM-1833 cells have not been examined, initial studies were performed to assess the effects of BGJ398 on viability of BoM-1833 cells. As shown in Fig 6A, treatment of cells with up to 100 nM BGJ398 does not affect cell viability as shown by an MTS assay. BGJ398 significantly reduces viability of cells that are dependent upon FGFR activity, the HC-11/R1 cell line [16], demonstrating that the inhibitor is functional (Fig 6B).

**Fig 7:**
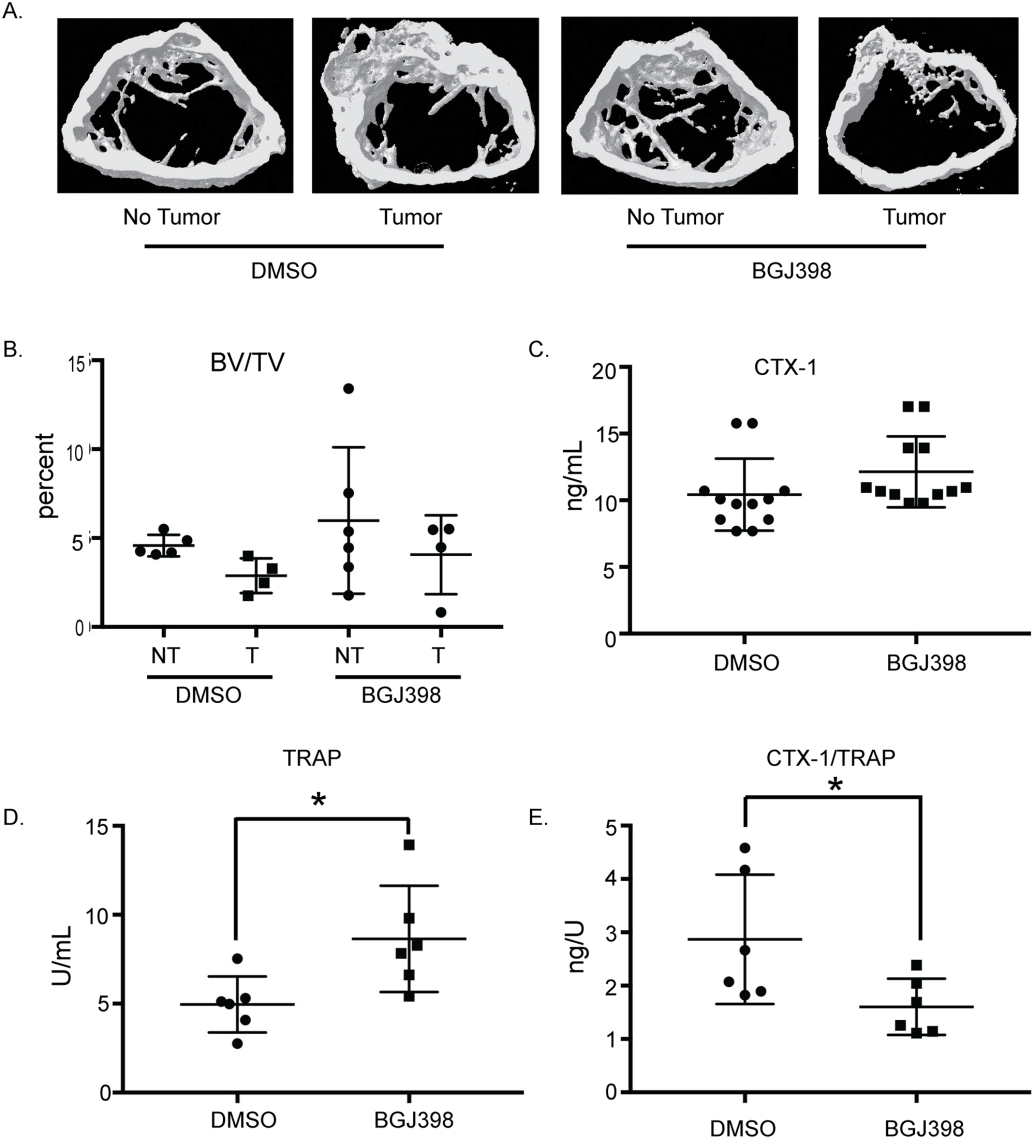
BGJ398 inhibits osteoclast activity in a bone metastasis animal model. (A) Micro-CT images of femurs from mice following direct femural injection of BoM-1833 cells. (B) Volumetric data quantifiying bone volume fraction (BV/TV). Sample size of mice DMSO, n = 9 (n = 5 without tumor and n = 4 with tumor) and BGJ398 n = 10 (n = 6 without tumor and n = 4 with tumor). (C-E) Serum was obtained from mice at euthanasia and subject to ELISAs. * p<0.05 comparing DMSO vs. BGJ398 treated mice.

Based on our *in vitro* results, we predicted that treatment of tumor bearing mice with BGJ398 would lead to a reduction in osteoclast activity. BoM-1833 cells were injected into the femurs of recipient athymic nude mice and treated with BGJ398. While no significant differences were observed in tumor size based on luciferase imaging (data not shown), micro-CT analysis (Fig 7A) revealed that the leg injected with tumor cells and treated with BGJ398 had significantly less bone destruction compared to the leg injected with tumor cells and treated with DMSO (compare T/DMSO, BV/TV = 2.877%, to T/BGJ398, BV/TV = 4.0677%, Fig 7B). Additionally, BGJ398 had no affect on the bone in the absence of tumor cells as indicated by the lack of significant bone destruction in the sham injected leg treated with BGJ398 compared to DMSO (compare NT/DMSO, BV/TV = 4.5784%) and NT/BGJ398, BV/TV = 5.9842% Fig 7B). Even though micro-CT images appear to show thicker cortical bone in the tumor bearing mice, measurements indicate that the presence of the tumor did not have a statistically significant impact on the cortical thickness at the diaphysis of the femora. Lastly we measured osteoclast activity and number with bone biomarkers serum collagen type 1 cross-linked C-telopeptide (CTX-1) and TRAP, respectively. There was no significant difference in osteoclast activity as measured by CTX-1 ELISA (Fig 7C). Measuring TRAP activity by ELISA has been demonstrated to correlate with osteoclast number in numerous mouse models [26–28]. We were unable to confirm the increase in osteoclast number by histological staining since femurs were only processed for micro-CT analysis. There was an increase in osteoclast number in the BGJ398 treated animals as indicated by the increase in TRAP detected in the serum (Fig 7D); however, upon comparison of activity per number of osteoclast (“resorption index”), we found that animals treated with BGJ398 had significantly less activity per osteoclast compared to DMSO treated animals and would explain the lack of a decrease in the BV/TV in the T/BGJ398 animals compared to the T/DMSO mice (Fig 7E).

## Discussion

These studies have focused on understanding the paracrine effects of tumor-derived FGFs on osteoclasts in the bone environment. Previously published studies have implicated FGF/FGFR signaling in the regulation of osteoclasts. Specifically, FGF2 has previously been shown to stimulate resorption of mature osteoclasts through activation of p42/44 MAP kinase [21]. FGF-6 and FGF-18 have also been shown to enhance osteoclast formation and promote absorption [9, 29]. In addition, deletion of FGFR1 in osteoclasts leads to reduced osteoclast formation and activity [30]. Because tumor cells are known to co-opt normal processes to enhance progression, we predicted that tumor cells that produce high levels of FGF ligands might act through the FGFR pathway to modulate osteoclast activity. Published studies have demonstrated that breast cancer cells are capable of producing FGF ligands, which can then feedback to the tumor cells and regulate proliferation and survival in an autocrine manner [12]. However, the effects of these soluble factors on the tumor microenvironment, in either the primary tumor site or in the metastatic site, are less well-understood.

Initial studies were performed to determine whether the FGF/FGFR axis contributes to normal osteoclast formation. We found a significant increase in expression of FGFR1 in osteoclasts during osteoclastogenesis. It was previously reported by Chikazu et al. [21] that FGFR1 was the only detectable FGF receptor expressed by mature osteoclasts; however, we detected highest level of FGFR1 expression around day 2 of RANKL stimulation before osteoclasts become mature, which occurs by day 4 in our culture system. The osteoclasts generated for the Chikazu et al. study were isolated from co-culture with osteoblasts where in our study the osteoclasts were generated by adding conditioned media containing M-CSF and recombinant RANKL. Additionally, in the Chikazu et al. study FGFR1 expression was not examined at any other time point during osteoclast differentiation. Therefore, it is not clear if they would have detected expression of FGFR1 at other time points during differentiation. Regardless of the pattern of expression, studies using a conditional FGFR1 knock-out model have demonstrated that FGFR1 is important for both differentiation and function of osteoclasts *in vivo* [30], providing a mechanism that can be co-opted by tumor cells to enhance osteolysis and promote growth in the bone environment. Analysis of FGF ligand expression in MDA-MB-231 cells revealed increased expression of ligands known to bind FGFR1, including FGF1, FGF19, FGF21 and FGF23 [24], suggesting that breast cancer cells express multiple potential regulators of FGFR1 activation that may act redundantly to induce FGFR signaling in osteoclasts.

Inhibition of FGFR activity during osteoclastogenesis led to reduced osteoclast size and a reduction in the number of nuclei per osteoclast, suggesting that FGFR activity is important for osteoclastogenesis. Whether this is due to increased sensitivity of osteoclasts to FGFs present within the serum in the growth media or whether this is due to autocrine production of FGFs by osteoclasts themselves remains to be determined. While exogenous administration of FGF ligands, including FGF-2 and FGF-9, have been linked to osteoclast regulation [21, 31, 32], production of FGFs by osteoclasts has not been extensively studied. Together, these results suggest an important role for FGFR during the process of osteoclastogenesis. Based on these findings, further studies were performed to determine whether the presence of soluble factors in conditioned medium obtained from tumor cells is capable of enhancing osteoclastogenesis in an FGFR-dependent manner. We observed a significant enhancement of osteoclastogenesis in response to tumor cell-derived factors in comparison with standard growth medium. Additionally, soluble factors from BoM-1833 cells enhanced resorption activity of osteoclasts, which was reduced upon inhibition of the FGFR pathway. Interestingly while we confirmed *in vivo* that FGFR inhibition may effectively reduce osteoclast activity in the bone metastatic site we did not observe a reduction in osteoclast differentiation *in vivo*. The difference between the *in vitro* and *in vivo* results suggests that there may be factors in the microenvironment that are capable of compensating for the loss in FGFR signaling in the context of osteoclast differentiation but these factors or other factors are not able to overcome the block to osteoclast activity.

ERK and PI3K signaling activation have been shown to be necessary for osteoclast activation and survival [33–36]. Additionally, loss of pAKT in osteoclasts leads to disruption of sealing zone formation via destabilization of microtubules [37]. Other studies with Src signaling an essential signaling pathway for osteoclast activity suggests that components of PI3K signaling pathway are downstream effectors of Src signaling pathway in regulating osteoclast activity [38]. p38 MAPK was upregulated by tumor derived factors and is essential for osteoclast differentiation but not activity [39]. Recently it was demonstrated that mice that are null for *Tgfbr2* in myeloid cells express decreased levels of bFGF in the tumor environment. The decreased bFGF resulted in less pERK activation but not pAKT activation in osteoclasts [40]. Our *in vivo* data suggest that loss of FGF activity by tumor cells is essential not for osteoclast differentiation but for osteoclast activity. Based on our findings *in vitro* in which tumor-derived factors activate ERK, Akt and p38 signaling pathways, which potentially impact both differentiation and activity, further studies are required to determine the relevant signaling pathways modulated by FGFR inhibition *in vivo*. It is feasible that soluble factors from other cell types within the microenvironment, and not modeled in the *in vitro* setting, are contributing additional factors that modulate the key signaling pathways and steer FGFR signaling to promote osteoclast activity rather than differentiation.

Stromal FGFR signaling has been implicated in tumor progression in other cancer models. For example, FGF-2 and FGF-8 have been implicated in promoting bone metastasis in renal cell carcinoma and prostate cancer, respectively [41, 42]. FGF-5 was identified as a gene in a bone metastatic signature that was generated from the MDA-MB-231 series of metastatic cell lines [15]. Furthermore, studies of prostate cancer have demonstrated cross-talk between FGF/FGFR signaling between tumor cells and osteoblasts in prostate cancer associated bone metastasis [43]. Treatment of preclinical models with dovitinib, a selective and clinically relevant FGFR inhibitor, demonstrated anti-tumor activity by modulating the tumor microenvironment [43]. Recently published studies using the MDA-MB-231 model demonstrated that FGFs produced by stromal cells can contribute to tumor cell proliferation in the bone [40]. These studies also demonstrated expression of FGFR1 in both tumor cells and osteoclasts within the metastatic environment [40]. Interestingly, we found that exposure of osteoclasts to tumor cell-derived factors led to altered expression levels of different FGFR isoforms. While FGFR1 isoforms were only modestly affected, changes in both FGFR2 isoforms and in FGFR3c were observed. Whether these changes in gene expression correlate with altered sensitivity of osteoclasts to different FGF ligands, or regulation of distinct gene targets within the osteoclasts remains to be determined. For these studies, we chose a cancer cell line that is not responsive to FGFR inhibition to assess the effects of FGFR inhibitors on the tumor microenvironment. While further studies are required to assess the efficacy of FGFR inhibition on growth of FGFR-dependent tumors, our results demonstrate that FGFR inhibition can target the tumor microenvironment. Combination therapies focused on targeting both the tumor cells and the stromal cells might lead to improved therapeutic responses by patients. Although FGFR inhibitors are currently being used in clinical trials for breast cancer patients, the specific effects of FGFR inhibitors on skeletal metastasis has not been examined. The importance of FGFR signaling in bone development and function warrants further investigation of this pathway in the context of breast cancer bone metastasis.

## Supporting information

S1 Fig Full length western blots. (A) Full length western blot analyzing FGFR1 expression during osteoclast differentiation. (B) Full length western blot analyzing β-tubulin expression as a loading control for osteoclast lysates.
(TIF)

S2 Fig Full length western blots of signaling pathways. (A) Full length western blot analyzed for (A) pp38, (B) total p38, (C) alpha-tubulin, (D) pERK, (E) total ERK, (F) alpha-tubulin and (G) pAKT signaling pathways in osteoclast lysates as shown in Fig 2.
(TIF)
