# Supplementary figures and images for: Breast cancer cell-derived fibroblast growth factors enhance osteoclast activity and contribute to the formation of metastatic lesions
Source: PLoS One. 2017 Oct 2;12(10):e0185736. doi: 10.1371/journal.pone.0185736 (PMC5624603; doi:10.1371/journal.pone.0185736)

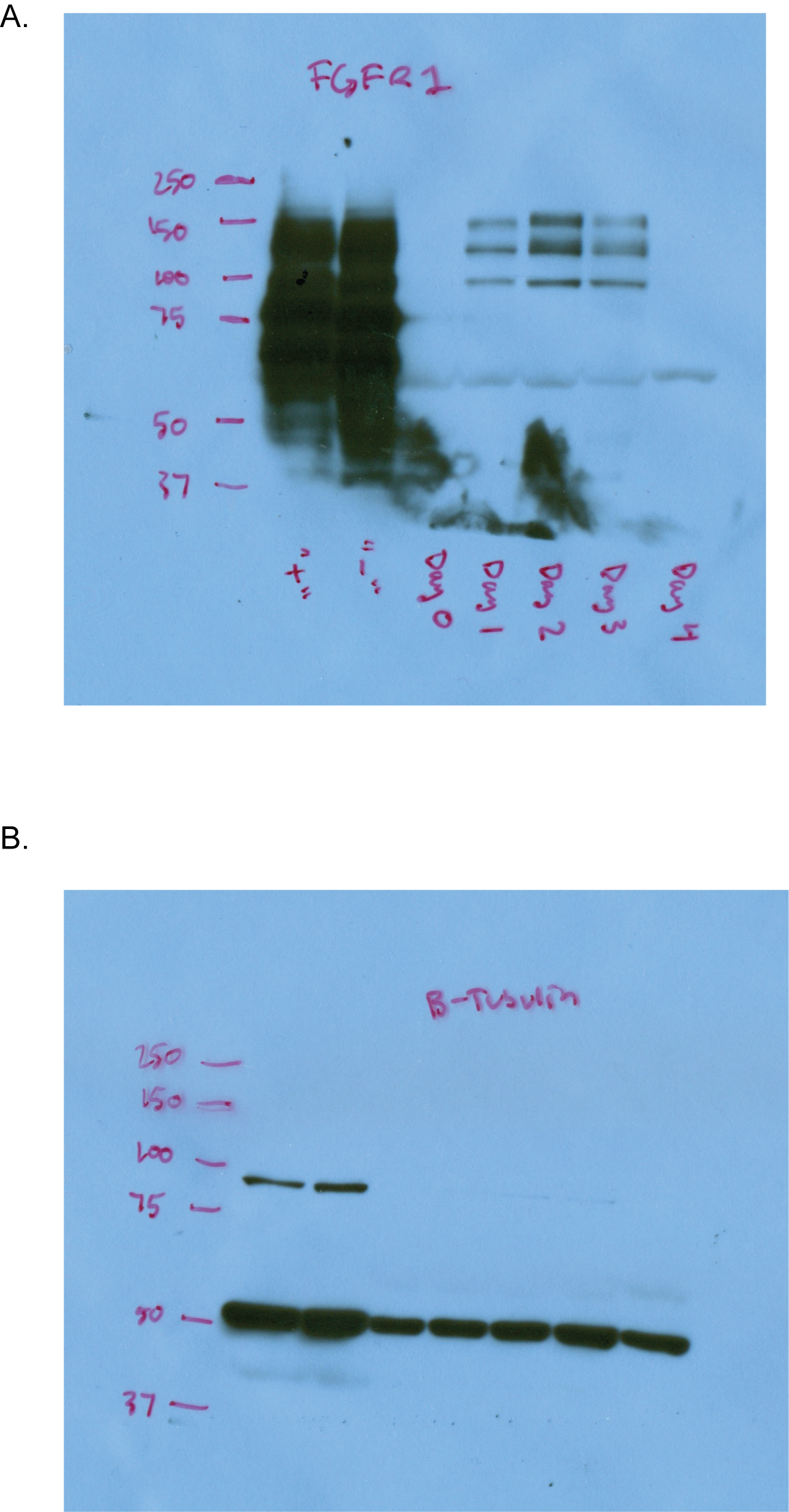

Supplement: S1 Fig — (A) Full length western blot analyzing FGFR1 expression during osteoclast differentiation. (B) Full length western blot analyzing β-tubulin expression as a loading control for osteoclast lysates. (TIF) [file pone.0185736.s001.tif]

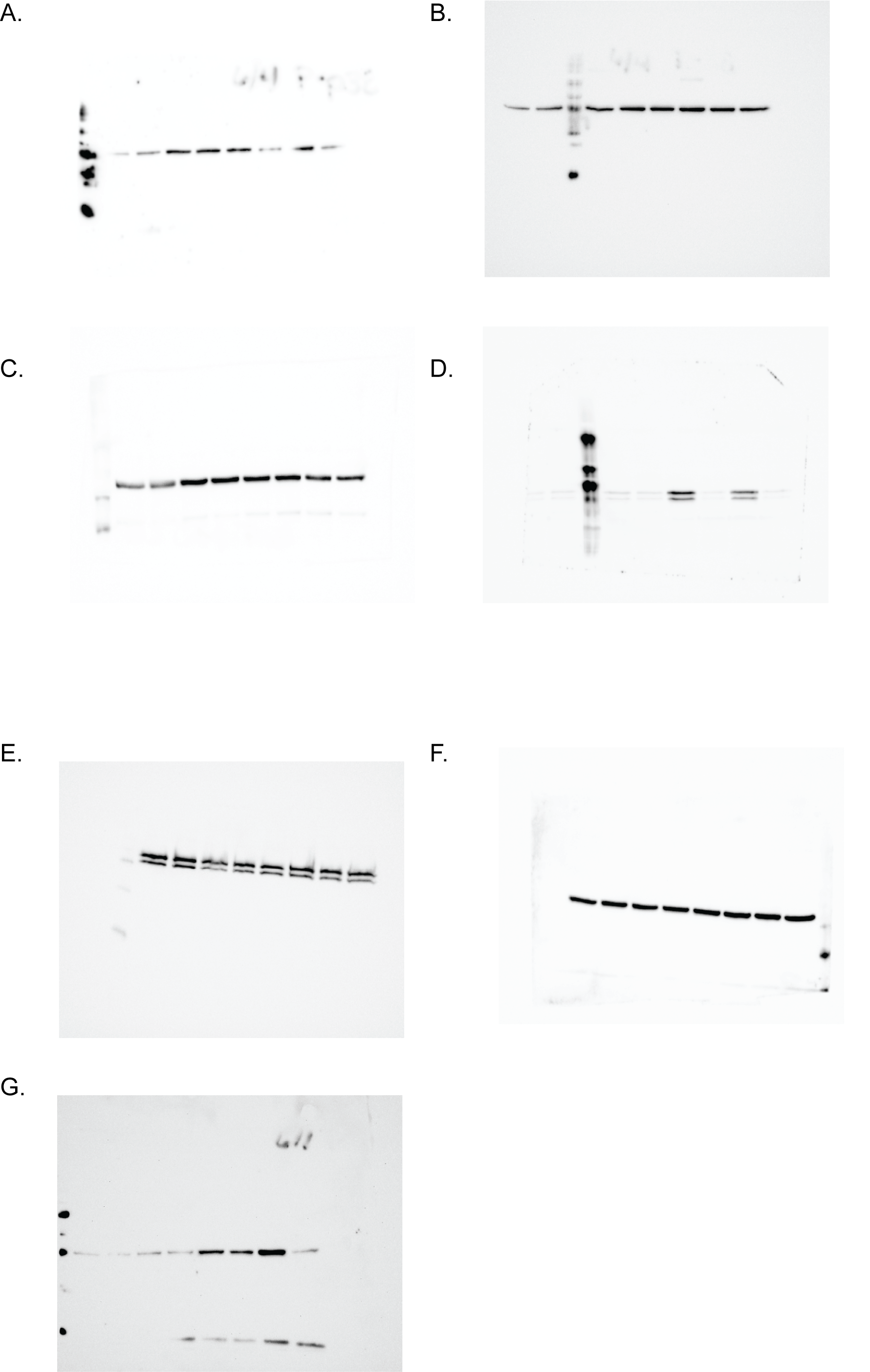

Supplement: S2 Fig — (A) Full length western blot analyzed for (A) pp38, (B) total p38, (C) alpha-tubulin, (D) pERK, (E) total ERK, (F) alpha-tubulin and (G) pAKT signaling pathways in osteoclast lysates as shown in Fig 2. (TIF) [file pone.0185736.s002.tif]
